# New Biomarkers in the Diagnosis and Prognosis of Dilated Cardiomyopathy: Pro-Resolving Lipids and miRNAs

**DOI:** 10.3390/cells14231916

**Published:** 2025-12-02

**Authors:** Rafael I. Jaén, Sergio Sánchez-García, María Fernández-Velasco, Irene Cuadrado, Beatriz de las Heras, Lisardo Boscá, Patricia Prieto

**Affiliations:** 1 Department of Metabolism and Cell Signaling, Biomedical Research Institute “Sols-Morreale” CSIC-UAM, 28029 Madrid, Spain; rafainigo@gmail.com (R.I.J.); t.sergio.sg@gmail.com (S.S.-G.); 2 Centro de Investigación Biomédica en Red de Enfermedades Cardiovasculares (CIBERCV), Instituto de Salud Carlos III, 28029 Madrid, Spain; mfvlorenzo@gmail.com; 3 Clinical and Translational Research Group in Cardiology, IdiPAZ/CIBER-CV, La Paz University Hospital, 28046 Madrid, Spain; 4 Pharmacology, Pharmacognosy, and Botany Department, Facultad de Farmacia, Complutense University, 28040 Madrid, Spain; icberrocal@ucm.es (I.C.); lasheras@ucm.es (B.d.l.H.)

**Keywords:** dilated cardiomyopathy, pro-resolving lipids, inflammation, biomarkers, correlation

## Abstract

Dilated cardiomyopathy is a major cause of heart failure and is one of the most common forms of cardiomyopathy worldwide. Although there has been significant progress in its clinical management, early diagnosis and precise prognosis remain challenging due to the lack of specificity in current biomarkers. As inflammation plays a key role in DCM, we determined the levels of systemic inflammatory markers and specific pro-resolving lipid mediators (SPMs) in a cohort of DCM patients. Our data show that the levels of lipoxin A4 significantly increased in DCM patients (343 + 75.1 pg/mL in controls vs. 482.2 ± 159.1 pg/mL in DCM patients), whereas the opposite was observed for resolving D1 (57.18 ± 32.68 pg/mL in controls vs. 38.55 ± 25.13 pg/mL in DCM patients). These results may indicate that SPMs could be considered new biomarkers related to the progression of this pathology. Moreover, since microRNAs (miRNAs) are also considered potential biomarkers at the molecular level, we conducted comprehensive miRNA expression profiling using a high-throughput array platform in our cohort. Of the differentially expressed miRNAs identified, we chose to focus on two that were significantly upregulated (miR378-3p and miR486-5p; more than two-folds) or downregulated (miR142-3p and miR328-3p < 20% and 40% vs. the control, respectively) in DCM patients, all of them strongly associated with inflammatory pathways. The selected miRNAs showed considerable potential as biomarkers, exhibiting statistical significance after ROC analysis. In fact, improved performance was observed when combining both miR142-3p and miR328-3p, using a LASSO regression model. However, we found no correlation between miRNAs and traditional inflammatory markers or SPMs ruling out the possibility to proposing them as combined biomarkers in this case. The heterogeneity of DCM leads to the need to identify new biomarkers that, either individually or in combination, may improve the prognosis of affected individuals. In our study, we have identified that some of the main SPMs can provide valuable information about disease progression, in addition to the combination of certain circulating miRNAs, which show promising prognostic values in our cohort. Thus, we have identified novel biomarkers that integrate inflammatory profiles with specific circulating miRNA expression patterns is an important step towards more targeted patient stratification in DCM. This approach can improve DCM diagnosis and prognosis, supporting the development of personalized treatments through a multi-parameter panel of biomarkers that can be measured in peripheral blood and used in routine clinical practice. Such a strategy can enable earlier treatment, resulting in better patient outcomes and quality of life.

## 1. Introduction

Cardiovascular diseases (CVDs) are the leading cause of death worldwide, responsible for approximately 18 million deaths each year, according to the WHO. Among CVDs, dilated cardiomyopathy (DCM) affects a significant number of patients, with an estimated prevalence of 1 in 250 adults [1]. DCM is characterized by the dilation of one or both ventricles, which results in impaired contractile capacity of the heart, as indicated by an ejection fraction (EF) of less than 40% [2]. The etiology is heterogeneous and several causes of DCM have been proposed, including mutations in various sarcomeric genes (e.g., *TTN*, *DES*, and *LDB3*), genes that are usually associated with severe arrhythmias (e.g., *LMNA*, *FLNC*, *PLN*, *TMEM43* and *RBM20*) [3,4] or with common metabolic disorders, such as diabetes and obesity [4]. Beyond genetic causes, also inflammatory conditions have been related to DCM development, like those associated with viral infections for parvovirus B19, herpesviruses, and SARS-CoV-2 [5].

This disease is progressive and often asymptomatic in the early stages, but it can lead to heart failure and, in some cases, death in later stages. Moreover, it is the primary cause of cardiac transplantation in young patients and contributes to many cases of sudden cardiac death [6]. Despite current knowledge and recent advances, particularly in the genetic aspects of this condition [7], it remains underestimated, mainly due to its high heterogeneity, which lowers the chances of a favorable prognosis for those affected. Therefore, further research is needed to improve early diagnosis and optimize patient treatment.

Several circulating biomarkers related to cardiac damage can be measured to characterize or diagnose the progression of DCM, helping clinicians in making informed treatment decisions. In this context, pro-BNP (N-terminal pro-brain natriuretic peptide) is a standard marker for cardiac damage, as its circulating levels increase after heart overload [8]. This marker is commonly used in the clinical diagnosis of CVDs as a predictor of adverse outcomes. In fact, values above 1000 pg/mL indicate adverse cardiovascular events and are strongly linked to a higher risk of mortality. Another important parameter is CRP (C-reactive protein), with elevated levels (≥5 mg/L) being indicative of a poor prognosis in cardiac disease, regardless of other hemodynamic parameters or even BNP levels [9,10]. However, despite the usefulness of these biomarkers, their specificity is limited, which restricts their predictive power. Therefore, measuring additional molecules and analyzing their combinations remains crucial to establish more effective combined biomarkers. In this regard, specialized pro-resolving lipid mediators (SPMs) are endogenous molecules that reduce inflammation and restore homeostasis [11]. SPMs can be produced by lipoxygenase enzymes from arachidonic acid (such as lipoxins) or from EPA and DHA (such as resolvins, maresins, and protectins). It is broadly known that their levels increase once the damaging stimulus has been removed, and they have the capacity to effectively stop inflammatory cascade, reducing the oxidative stress and promoting tissue regeneration. Thus, SPMs and their derivatives have demonstrated protective effects in animal models of cardiac disease, such as myocarditis [12], diabetic cardiac dysfunction [13], and even myocardial infarction [14]. Among SPMs, lipoxin A_4_ (LXA_4_) was the first described in the 80s and it has been broadly described its pro-resolutive role modulating the immune system in order to adequately finalize inflammation and recover tissue homeostasis [15]. Furthermore, high levels of LXA_4_ have been associated with a lower risk of ischemic events in patients with acute myocardial infarction [16]. More recently, reduced serum RvD1 levels have been linked to worse outcomes in patients with aneurysmal subarachnoid hemorrhage [17] or those experiencing ST-segment elevation myocardial infarction [18]. Therefore, SPM levels could help determine the inflammatory profile of DCM patients and potentially improve the prognosis and diagnosis of DCM.

Beyond the inflammation-related markers traditionally used for patient classification, recent studies suggest other potential biomarkers to improve patient stratification. These molecules, which should be detectable in the bloodstream, provide valuable additional information to better characterize disease profiles and enhance personalized treatment strategies. Among them are microRNAs (miRNAs), endogenous non-coding RNA molecules that have become promising prognostic targets. Few works have explored the interaction between the SPMs and the miRNAs, emphasizing how some SPMs regulate miRNAs in acute inflammation and in the cardiovascular system [19,20]. It has been proposed that this interaction could play a key role in reducing the inflammatory response and promoting resolution [21].

Many circulating miRNAs have recently been quantified and proposed as biomarkers for prognosis or early diagnosis in various diseases, including cardiac damage [22]. Specifically, some miRNAs are already linked to DCM and are associated with the development of cardiac fibrosis, hypertrophy, or dysfunction [23,24]. Significant variability exists among cohorts regarding which miRNAs are notably altered in DCM patients, mainly due to a lack of standardized protocols. This underscores the urgent need for continued research into miRNA levels in DCM patients and their relationship with other cardiac damage markers.

Since the origin of DCM involves both genetic factors related to cardiac sarcomeric function and conditions characterized by chronic inflammation—including metabolic syndrome—as well as infectious processes that cause acute inflammation, especially viral infections, the biomarkers associated with DCM will vary depending on the underlying pathology. In this context, this study identified specific signatures involving SPMs and changes in certain circulating miRNAs. Therefore, combining different types of molecules has the potential to create a more accurate biomarker panel for early clinical diagnosis and prognosis of this condition, ultimately leading to better patient outcomes. In fact, the potential relationship between miRNAs and SPMs as biomarkers in the context of dilated cardiomyopathy has not been previously addressed, supporting the novelty of the present study.

## 2. Material and Methods

### 2.1. Human Samples

Clinical samples and related data from patients diagnosed with DCM (including BNP determinations) were managed and provided by Biobanco La Fe (PT17/0015/0043) or Basque Biobank (Available online: www.biobancovasco.org, accessed on 28 November 2025). They were processed following standard operating procedures with approval from the ethical and scientific committees. Sample processing was conducted according to the standard procedures of both institutions. Due to the limited availability of female samples, the study population was restricted to male participants. Healthy male controls were recruited from the Blood Donation Service at La Paz Hospital after giving informed consent. All procedures complied with Spanish biomedical research regulations (Law 14/2007-3) and followed the principles outlined in the 2013 Declaration of Helsinki.

### 2.2. CRP Determination

CRP concentrations were measured using an enzyme-linked immunosorbent assay (ELISA) kit (Enzo Life Sciences, Farmingdale, NY, USA, #ENZ-KIT102) according to the manufacturer’s instructions. Briefly, 100 µL of human serum per sample was incubated in duplicate with CRP conjugate and the corresponding antibody at room temperature for 2 h. After washing, the reaction was developed with horseradish peroxidase (HRP) and TMB substrate, and absorbance was read at 450 nm.

### 2.3. LXA_4_ and RvD1 Quantification

LXA_4_ levels in human serum were measured using an ELISA kit (Neogen, Lexington, KY, USA, #407010). Before analysis, lipid enrichment was performed with C18 Sep-Pak cartridges (Waters Corporation, Milford, MA, USA, #WAT023501). RvD1 concentrations were determined using an ELISA kit from Cayman Chemical (Ann Arbor, MI, USA, #500380). Both determinations were performed following the manufacturer’s instructions. These assays are validated in various human biofluids, including serum, plasma, and urine.

### 2.4. miRNA Profiling in Human Serum via TLDA

Three healthy male donors (over 50 years old) and three age-matched DCM patients from Biobanco La Fe were selected from the collected serum samples. Total RNA, including miRNAs, was extracted using the MagMAX™ mirVana™ Total RNA Isolation Kit (Thermo Fisher Scientific, Waltham, MA, USA, #A27828). Complementary DNA (cDNA) synthesis was performed with the TaqMan Advanced miRNA cDNA Synthesis Kit (#A25576). miRNA expression profiling was conducted using TaqMan Advanced miRNA human serum/plasma cards (#A34717 Thermo scientific) at the IIBM Sols-Morreale Genomic Service on an Applied Biosystems 7900 HT FAST Real-Time PCR System. These cards are specifically designed to detect 188 miRNAs in human serum or plasma samples including three miRNAs as controls, hsa-miR-16-5p as endogenous and cel-miR-39-3p, ath-miR159 as exogenous. Data analysis was performed using the ΔΔCt method with GraphPad Prism version 8.0.2 software (Dotmatics, Boston, MA, USA), to obtain the corresponding *p* value. These results were represented as volcano plots and bar graphs where green dots or bars indicate upregulated miRNAs and red dots and bars represent downregulated miRNAs (|log2FC| ≥ 1). Then, significantly regulated miRNAs were categorized by functional pathways based on expression changes using the MiRWalk database and represented in a graph bar regarding their *p* value.

### 2.5. miRNA Validation by qPCR Analysis

For validation, miRNA extraction and reverse transcription were performed as described in the section above. Quantitative PCR (qPCR) was carried out using TaqMan Advanced miRNA Assays (Table 1) and the TaqMan Fast Advanced Master Mix (#4444556). Relative expression levels were determined using the ΔΔCt method.

### 2.6. Statistical Analysis

All statistical analyses were performed using GraphPad Prism version 8.0.2 software (Dotmatics, Boston, MA, USA). First, we applied the Shapiro-Wilk test to all data sets to determine if they follow a normal distribution. For those that passed the normality test, parametric statistical test was applied: unpaired *t*-test to compare two groups and one-way ANOVA followed by Tukey’s post hoc test to compare multiple groups. To those data that did not follow a normal distribution, we used the non-parametric Mann–Whitney test. The specific statistical analysis and corresponding *p*-values are shown in each figure.

To analyze multiple miRNAs as diagnostic biomarkers, a nested cross-validation framework with L1-regularized logistic regression (LASSO) was applied. Samples with any missing values were excluded at the beginning of the analysis. Firstly, nested cross-validation was implemented, with an outer loop of 5 stratified folds for model evaluation, and an inner loop of 3 stratified folds for hyperparameter tuning. In the inner loop, GridSearchCV was used to identify the optimal regularization strength (C parameter) across 10 logarithmically spaced values from 10^−3^ to 10^2^. The optimal model was refit on the training portion of each outer fold and used to generate predictions for the corresponding test fold. The model’s predictive performance was assessed via the ROC/AUC analysis and classification accuracy on the outer test folds. To evaluate feature stability, the frequency with which each miRNA was assigned a non-zero coefficient across the five outer fold models was recorded. This provided an empirical measure of feature robustness and importance. Thus, the LASSO model retained two miRNAs (miR142-3p and miR328-3p), while the other two (miR378a-3p and miR486-5p) were shrunk to zero. For this reason, the analysis was redone using only the two significant miRNAs in order to maximize the sample size, as some patients had missing values in any of the now discarded miRNAs.

Pearson correlation analysis examined the relationships between the expression levels of differentially expressed miRNAs and clinical variables such as age, CRP, left ventricular ejection fraction (LVEF), and NT-proBNP. A *p*-value < 0.05 was considered statistically significant.

## 3. Results

To conduct this study, we recruited a cohort of 21 DCM patients and 26 healthy controls, both groups within the same age range, averaging around 50 years old (Table 2). Regarding the clinical data obtained from the biobank sources, this cohort had a mean LVEF of less than 44%, with 62% (13/21) showing values below 20%. Moreover, DCM patients showed elevated pro-BNP levels, reaching an average of 4.9 ng/mL (±3.4), which may indicate cardiac damage, supporting the diagnosis of DCM.

**Figure 1:**
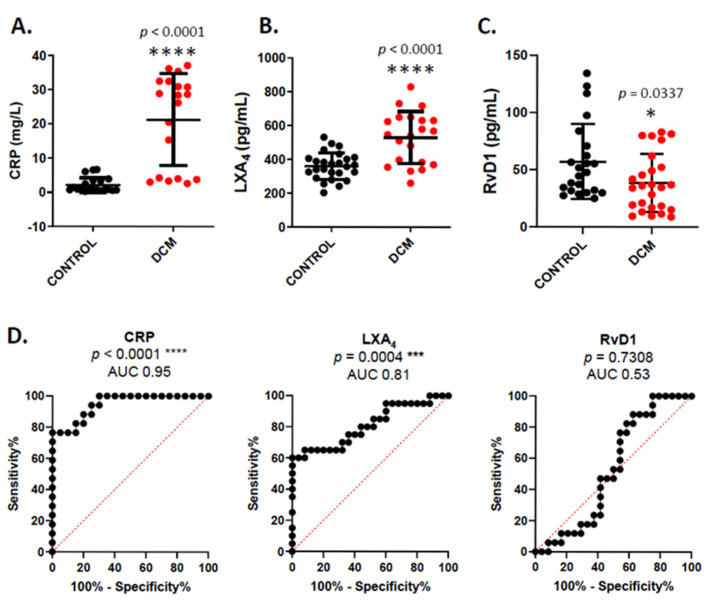
Determination of serum levels of (**A**) C-reactive protein (CRP), (**B**) lipoxin A4, and (**C**) resolvin (D1) in healthy controls and DCM patients. Mann–Whitney test was used in (**A**,**C**), and unpaired *t*-test was used in (**B**). * *p* ≤ 0.05 and **** *p* ≤ 0.0001 versus the corresponding controls. (**D**). ROC/AUC analysis of CRP, LXA_4_ and RvD1. Graphs show the ROC curve of each parameter, which reflects their performance as predictors. AUC and *p*-values are displayed. *** *p* ≤ 0.001; **** *p* ≤ 0.0001 versus random prediction (AUC = 0.5).

Since DCM is an inflammatory disease, we analyzed the serum levels of various inflammatory markers in healthy donors and the DCM cohort (Figure 1). Systemic concentration of CRP is a key parameter for assessing inflammation in DCM patients, with higher levels indicating a worse prognosis. As expected, controls have a mean value of 2.12 mg/L (±0.48), which increased tenfold to 21.28 mg/L (±3.08) in DCM patients (Figure 1A).

**Figure 2:**
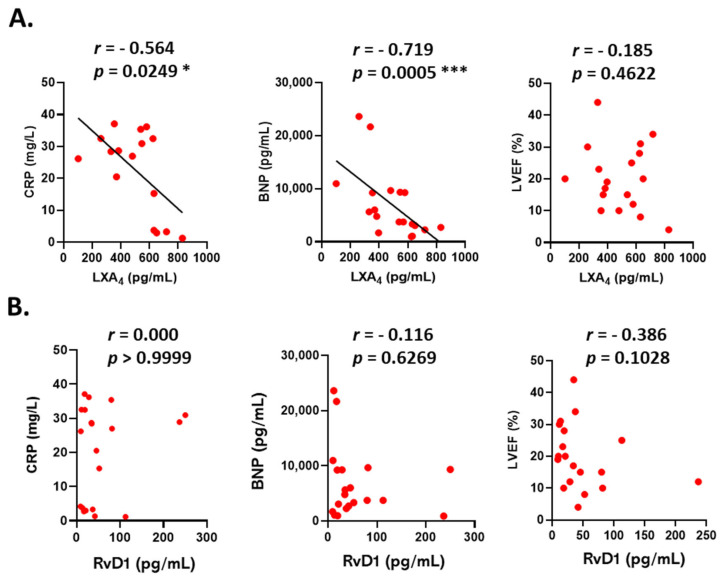
Correlation analysis between serum levels of C-reactive protein (CRP), B-type natriuretic peptide (BNP) and left ventricular ejection fraction (LVEF) with LXA4 (**A**) or RvD1 (**B**) systemic levels in DCM patients. Each red dot corresponds to an independent patient. The values shown in each graph indicate the corresponding Pearson’s r ((**A**): LVEF) or Spearman’s r ((**A**): CRP, BNP; (**B**): CRP, BNP, LVEF) correlation coefficients and *p*-values * *p* ≤ 0.05; *** *p* ≤ 0.001.

After analyzing this classic inflammatory marker, we evaluated the levels of SPMs, which are crucial during the resolution phase of inflammation. We observed that LXA4 levels were significantly higher in DCM serum compared to the control group, while RvD1 levels were lower (Figure 1B,C). In fact, when we performed an AUC/ROC analysis to assess the diagnostic potential of both CRP and these SPMs as possible biomarkers in DCM, we detected a significant predictor capacity of CRP and LXA4 (Figure 1D). Next, to explore the potential relationship between pro-inflammatory and pro-resolving mediators in these serums, we conducted a correlation analysis between SPM levels and the pathological markers CRP, BNP, and LVEF (Figure 2). We found that LXA4 was inversely correlated with CRP and BNP levels. The same pattern was observed with LVEF, although it was not statistically significant (Figure 2A). However, the correlations involving RvD1 were not statistically significant (Figure 2B). This lack of correlation may indicate that SPMs are regulated independently from other traditional inflammation markers, especially at the systemic level.

**Figure 3:**
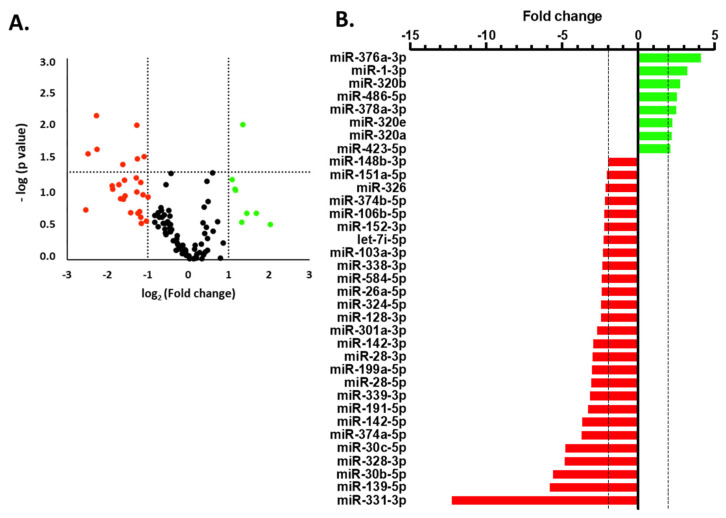
Determination of miRNA levels in the DCM cohort by TLDA. (**A**). Volcano plot showing the distribution of miRNAs in DCM patients. Green dots indicate upregulated miRNAs and red dots represent downregulated miRNAs (vertical dashed lines, |log2FC| ≥ 1). Horizontal dashed lines mark the significantly modified miRNAs (−log(*p*-value) ≥ 1.3) (**B**). The most extensively modified miRNAs are shown as a bar graph. Vertical dashed lines indicate |FC| ≥ 2.

**Figure 4:**
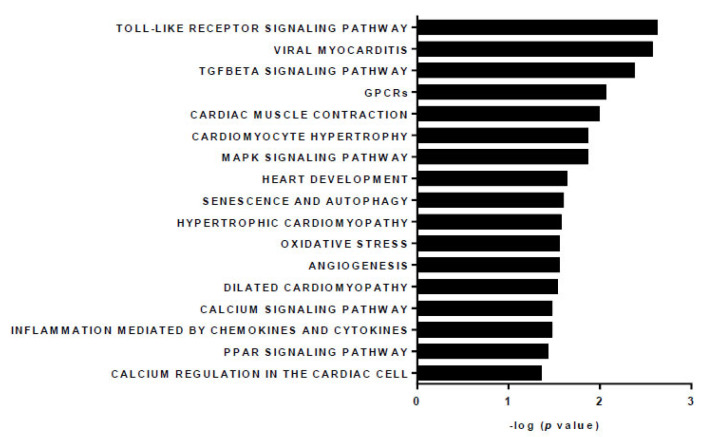
Top signaling pathways related to the miRNAs that were significantly modulated in the DCM cohort *versus* the healthy cohort.

In addition to analyzing inflammatory markers, we aimed to explore potential differences in miRNAs within the DCM cohort, as their regulation is crucial for the development of various pathologies, and several have already been proposed as biomarkers for DCM [24]. Therefore, we initially decided to conduct an array of miRNAs in serum samples from three DCM patients and three healthy male donors, all of whom were over the age of 50 (Figure 3). To abord it, we used miRNAs Taqman^®^ Advanced Cards, that are specifically designed to detect the circulating levels of 188 miRNAs in biological samples of human serums. miRNA profiling revealed significant modulation of various specific miRNAs in DCM patients; 35 were modified more than 2-fold (8 upregulated and 27 downregulated). After identifying which miRNAs are altered in DCM patients, we examined their enrichment in functional clusters using bioinformatics tools and summarized the most relevant pathways in a bar graph (Figure 4). As a result, the significantly changed miRNAs in DCM can be grouped into 17 functional categories, among which we can highlight enriched pathways related to inflammation (TLR signaling, TGFβ pathway, GPCRs, MAPKs, PPARs, etc.), as well as several cardiac-related clusters such as viral myocarditis, cardiac muscle contraction, heart development, and hypertrophic and dilated cardiomyopathy, among others.

**Figure 5:**
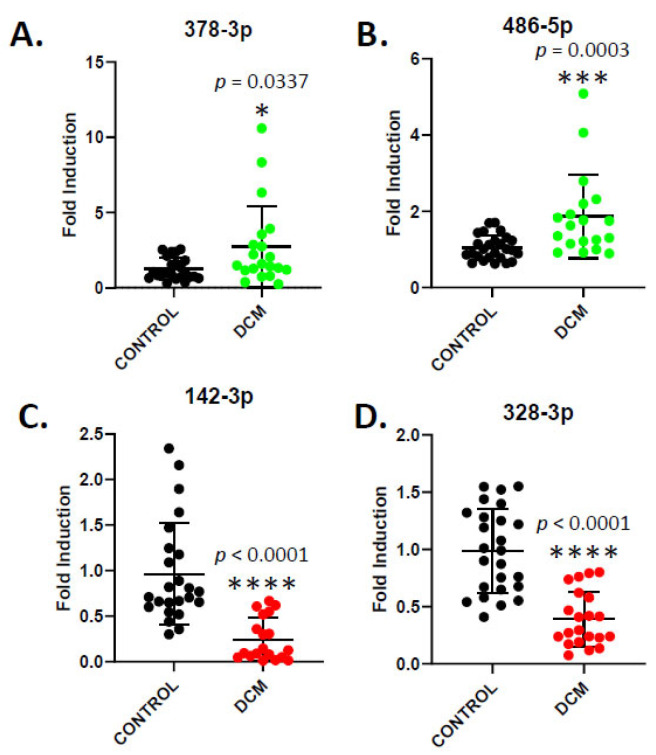
Determination of the levels of selected miRNAs (miR-378-3p in (**A**), miR-486-5p in (**B**), miR-142-3p in (**C**) and miR-328-3p in (**D**)) in healthy donors and DCM patients by RT-qPCR. Each dot corresponds to a single individual. Exact *p*-values are displayed, calculated with Mann–Whitney test. * *p* ≤ 0.05; *** *p* ≤ 0.001; **** *p* ≤ 0.0001 *versus* control.

To verify these findings, we selected two upregulated (miR-378a-3p, miR-486-5p) and two downregulated (miR-142-3p, and miR-328-3p) miRNAs that had already been shown to influence cardiac disease in published studies in the field of DCM [24]. Thus, miR-378a-3p has been identified as a biomarker for several cardiovascular diseases and acts as a cardioprotective factor [25], and miR-486-5p has been associated with acute myocardial infarction [26]. Regarding miR-142-3p, a decrease has been observed in heart failure and during the progression to fibrosis [27], and miR-328-3p appears to exert protective roles on endothelial cells [28,29]. We then measured the levels of these selected miRNAs in the entire cohort, using Taqman technology and miR-1-3p as a control, to verify the results obtained in the array (Figure 5).

**Figure 6:**
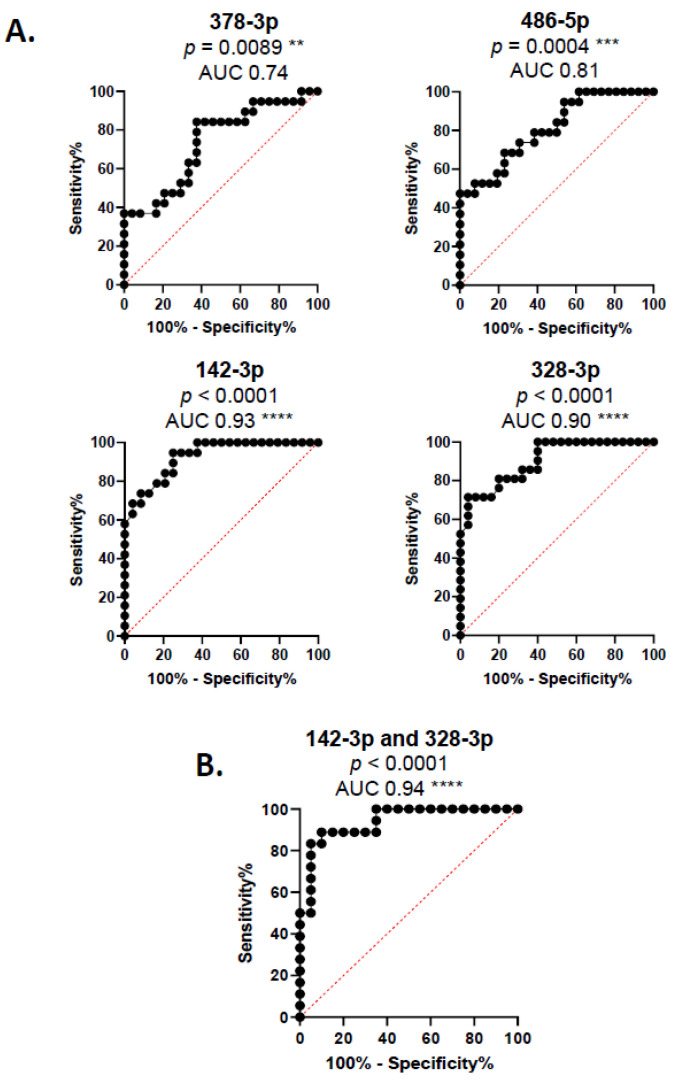
ROC/AUC analysis of selected miRNAs. The graphs show the ROC curves of (**A**) individual miRNAs or (**B**) the combination of miR142-3p and miR328-3p, using a nested cross-validation LASSO regression model. AUC values and *p*-values are displayed. ** *p* ≤ 0.01, *** *p* ≤ 0.001, **** *p* ≤ 0.0001 versus random prediction (AUC = 0.5).

Our previous results were confirmed, as miR-378a-3p and miR-486-5p were significantly upregulated in our samples, decreasing miR-142-3p and miR-328-3p levels. Next, we performed an AUC/ROC analysis to assess the diagnostic potential of these miRNAs as possible biomarkers in DCM (Figure 6). All evaluated miRNAs were statistically significant, indicating their ability to distinguish DCM patients from controls, highlighting their potential as biomarkers (Figure 6A). Additionally, we tested whether combining multiple miRNAs could provide a more accurate prediction using a multivariate LASSO model. The combination of miR-142-3p and miR-328-3p was a stronger predictor than any other combination, including individual miRNAs, as the AUC was shown to be 0.94 (Figure 6B).

**Figure 7:**
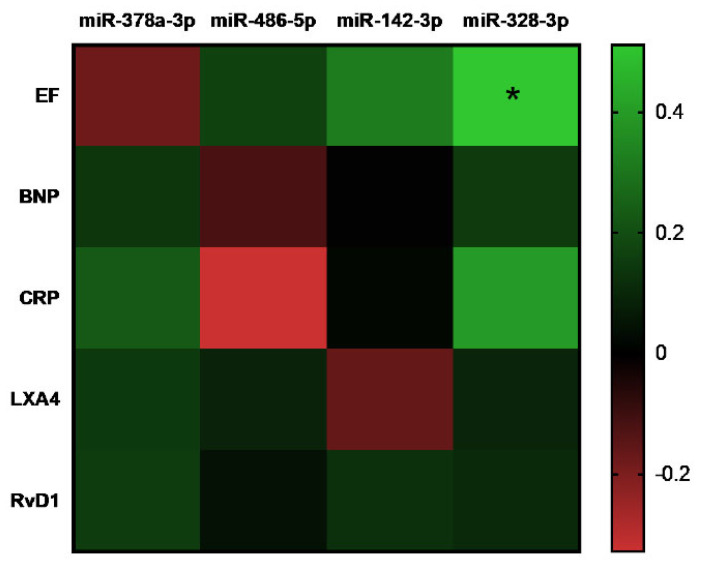
Correlation matrix of the selected miRNAs *versus* EF, BNP and CRP in DCM patients. Color intensity correlates with a higher Pearson’s |*r*| value. * *p* ≤ 0.05.

Lastly, we created a combined correlation matrix to explore possible relationships among all systemic and miRNA biomarkers (Figure 7). Unfortunately, only a significant correlation was found between miR-328-3p and LVEF, while the others did not reach statistical significance.

## 4. Discussion

DCM remains a common cardiac condition, with early diagnosis being difficult due to high variability in clinical progression and treatment response among patients. Recently, there has been increased interest in enhancing cardiovascular diagnostic and prognostic tools through the combined analysis of systemic biomarkers easily measured in clinical settings. Instead of relying on single indicators, integrating multiple independent molecular parameters may offer a more comprehensive understanding of disease progression, especially in conditions like DCM. In this study, we aimed to go beyond typical inflammatory markers such as BNP or CRP. We also measured specialized pro-resolving mediators LXA_4_ and RvD1 to identify differences between groups that could improve diagnostic accuracy. We found that DCM patients have significantly higher circulating levels of LXA_4_ than controls (483.3 pg/mL vs. 374.3 pg/mL), indicating their bodies’ attempt to resolve inflammation. In fact, the ROC curve shows a significant relevance of this SPM as a biomarker. This aligns with findings in other diseases like COVID-19 [30] and preeclampsia [31], where circulating levels of these SPMs were markedly higher compared to healthy individuals. Notably, LXA_4_ levels in these studies were within the same range as observed here [32]. Conversely, RvD1 levels were lower in our DCM group, decreasing from an average of 50.36 pg/mL in healthy donors to 38.55 pg/mL in patients. Multiple studies have reported reduced circulating RvD1 levels in various diseases, including arterial hypertension [33], rheumatoid arthritis [34], and ulcerative colitis [35]. Similar reductions have been documented in cardiovascular conditions such as hemorrhagic subarachnoid aneurysm [17], ST-segment elevation myocardial infarction [18], and acute coronary syndrome [36]. These findings suggest that inflammatory diseases are characterized by overactivation of pro-inflammatory pathways and an imbalance in the resolution phase of inflammation. This imbalance leads to impaired production of pro-resolving mediators over time, hindering the body’s ability to halt inflammation and restore tissue homeostasis. Beyond measuring each mediator individually, we also investigated whether their correlations could provide diagnostic or prognostic insights in DCM. We found a significant inverse correlation between LXA_4_ levels and both CRP and BNP. Therefore, although LXA_4_ levels are elevated in DCM patients, those with greater systemic inflammation exhibit lower LXA_4_, indicating a potential link between impaired resolution mechanisms and markers of systemic inflammation and cardiac stress. Regarding CRP, this inverse relationship had been previously documented in various cardiovascular studies [37,38]. In fact, the expression of CRP has been associated with decreased LXA_4_ synthesis in foam cells in a rabbit model of atherosclerosis [32].

Several circulating miRNAs have been identified as potential biomarkers that play essential roles in common diseases [25,39,40,41] and in DCM, some of which align with our panel [24]. Therefore, we examined a panel of miRNAs that enables the identification of the main circulating human miRNAs with known roles in cardiac pathophysiology in our DCM patient cohort. By exploring possible relationships among these molecules, we aimed to develop a new biomarker signature for DCM that could complement the previous ones, enabling earlier diagnosis and improving both short-term and long-term clinical outcomes. Consistent with previous findings, we observed significant changes in several miRNAs linked to inflammation-related pathways such as TLRs, myocarditis, TGFβ, and GPCR signaling, as well as those involved in cardiac functions like muscle contraction and heart development. Interestingly, the main specific receptor for LXA_4_, known as ALXR, belongs to the GPCR family [42], so it is not surprising that this signaling pathway appears to be modulated in the study. Regarding the others, although these are very general signaling pathways, there is specific evidence that miR-378 is capable of modulating the MAPK pathway [43] whereas miR-486-5p, miR-328-3p or miR-142-3p have been associated with PI3K/Akt [44,45,46]. Among the miRNAs altered in DCM, we selected two upregulated and two downregulated sequences based on their statistical significance and prior association with DCM. Notably, miR378-3p has been connected to heart disease in numerous studies. It has been proposed as a key player in cardiovascular diseases, generally providing protective effects [25]. Specifically, miR-378 has been shown to protect cardiomyocytes from doxorubicin-induced cell death [47], and its expression correlates with a reduction in cardiomyocyte hypertrophy [48,49]. However, some researchers report that this miRNA can also be harmful by increasing cardiomyocyte sensitivity to apoptosis [50], and elevated levels of miR-378 have been suggested as early biomarkers for diabetic cardiomyopathy [51]. In line with this, we observed an increase in miR-378-3p in our DCM cohort. This suggests that this miRNA is a suitable biomarker for some cardiac pathologies, irrespective of its beneficial or prejudicial effects. However, further studies are needed to clarify its specific effects in DCM. Similar effects have been observed for the other upregulated miRNA, which exhibits an even stronger protective role. It has been reported that miR-486-5p protects against cardiomyocyte apoptosis in different models, helping to reduce cardiac injury and improve cardiac function [52,53,54,55,56]. Interestingly, this miRNA also promotes angiogenesis after myocardial infarction, which has been linked to preventing adverse remodeling and supporting tissue repair, ultimately reducing the risk of heart failure [57,58]. Therefore, the increase in miR-486-5p in DCM may reflect a compensatory mechanism for the harmful effects of the disease. Conversely, we observed lower levels of miR-142-3p in DCM patients, consistent with previous findings from Nair et al. [59]. This miRNA is considered protective because its overexpression reduces inflammation, prevents hypertrophy, and improves mitochondrial function [60,61]. Thus, decreased levels could be damaging to the DCM heart. Additionally, high levels of miR-328-3p have been linked to an increased risk of atrial fibrillation [61], suggesting that lowering its levels might benefit DCM hearts. In fact, this miRNA increases cardiomyocyte apoptosis and fosters myocardial infarction in rats [62].

Our next step was to assess whether these miRNAs could serve as diagnostic markers, regardless of their potential beneficial or harmful effects. Our data show that the four selected miRNAs can distinguish between DCM and healthy controls. Notably, both miR142-3p and miR328-3p exhibited AUC ≥ 0.9, indicating that their ability to discriminate between the two groups is excellent. Furthermore, the combination of miR-142-3p and miR-328-3p exhibited superior performance compared to individual miRNAs, achieving an AUC value of 0.94. This indicates that measuring these two miRNAs together could provide a highly effective diagnostic tool in clinical practice. Although we found a correlation between miR-328-3p and LVEF, no other significant associations between miRNAs and circulating inflammatory or resolution biomarkers were observed. This may be due to the limited sample size or the heterogeneity in DCM progression among patients in this cohort. Moreover, biomarker levels vary considerably throughout the course of the disease, from a pro-inflammatory to a resolution phase, meaning that possible correlations may show up depending on the moment when the sample is taken. Nevertheless, a direct correlation exists between reduced miR-328-3p levels and the ejection fraction in DCM patients. In conclusion, we demonstrated that LXA_4_ levels and at least four miRNAs differentially expressed in DCM patients versus healthy individuals effectively distinguish between the groups. Notably, combining miR-142-3p and miR-328-3p yielded an enhanced predictive capacity. These findings suggest that assessing SPMs and miRNAs alongside traditional biomarkers could improve diagnostic accuracy.

## 5. Conclusions

Based on the results presented in this work, it can be suggested that differences in biomarker profiles in DCM may be related to the primary cause of the disease. This could facilitate faster patient stratification and the development of targeted therapeutic interventions to prevent DCM progression.

## 6. Limitations of the Study

The cohort analyzed included only men due to the difficulty in obtaining female samples. Nevertheless, the patients had well-established diagnoses, and the results, which integrated various markers, demonstrated strong robustness and supporting their application in longitudinal studies of disease progression and prognosis in these patients. This is particularly significant because diagnosis is complex and often delayed, usually occurring after the disease has already established itself. Even so, it would be interesting to include female samples in future studies in order to deep into the knowledge of signaling pathways in them.

## Figures and Tables

**Table 1:** Taqman Advanced miRNA Assay sequences.

miRNA	miRBase Accession Number	Mature miRNA Sequence
Hsa-miR-142-3p	MIMAT0000434	UGUAGUGUUUCCUACUUUAUGGA
Hsa-miR-328-3p	MIMAT0000752	CUGGCCCUCUCUGCCCUUCCGU
Hsa-miR-378a-3p	MIMAT0000732	ACUGGACUUGGAGUCAGAAGGC
Hsa-miR-486-5p	MIMAT0002177	UCCUGUACUGAGCUGCCCCGAG

**Table 2:** Demographic (**A**) and clinical (**B**) data for healthy donors and dilated cardiomyopathy (DCM) patients. N corresponds to the number of people included in each group.

**A**		
	**Healthy donors** **(N = 26)**	**DCM** **(N = 21)**
Age, mean ± SD (years)	50.23 ± 9.58	53.4 ± 13.45
Age, min–max (years)	29–65	19–67
**B**		
**DCM**		
PRO-BNP (pg/mL)	4908.1 ± 3390	
LVEF	≤20%	13
	20–44%	7
	NOT DATA	1

## Data Availability

For ethical reasons, additional data from the cohort will be available on request.
